# Supplementary material for: Performance evaluation of nano-graphene lubricating oil with high dispersion and low viscosity used in diesel engines
Source: PLoS One. 2024 Aug 16;19(8):e0307394. doi: 10.1371/journal.pone.0307394 (PMC11329151; doi:10.1371/journal.pone.0307394)
Supplement: Attachment — Submitted filename: Comments.docx [file pone.0307394.s001.docx]

1. Though introduction section clearly highlights the current study's novelty. however, a justification for using graphene as an additive over other nano particles has not been provided.
2. In Line 95, what is “0#” represents?
3. In Line 100, Author claims that “The nano-graphene was chemically modified by oleic acid and stearic acid”. Whereas nano-graphene should not be reacted with acids.
4. In Line 102, Author claims that “2g stearic acid and 3 g oleic acid were then added into them” It is not cleared about the purpose of adding the acids. Again, why only these two particular types of the acids are added?
5. Comparing Table 2 and 3: Density of Diesel is 836 kg/m3 and density of PLO is 0.893 kg/ m3. If so, then how the flash point of PLO is significantly higher than diesel.
6. Line 208: “The experimental fuel were two kinds of mixed diesel fuel, respectively, diesel added with 0.5% mass fraction of PLO and diesel added with 0.5% mass fraction of MGL25.” Elaborate the process if possible.
7. Line 215: “After the engine idled for half an hour, the formal experiment began when the lubricating oil temperature reached about 80°C.” already given in line Line:157.
8. Table 10 represents the engine emissions where as the unit given in g/KW-h. How that can be possible?
9. Moreover, From the table 10 it is found NOx emission is significantly lower for MGL25, whereas Fig. 10 represents fuel consumption for MGL25 is lower throughout the speed variation, that means higher efficiency of the engine. Why this controversy occurs?
10. The quality of Fig.11 should be improved.
11. In conclusion claimed about the good antifriction effect of nano-graphene lubricant over generally used lubricant. Whereas no comparison of tribological behaviours between these two lubricants have not found.
